# Expression of fibroblast growth factor 23 (FGF23) and αKlotho in two commercial laying hen strains fed with and without dietary mineral P supplements before and after the onset of the laying phase^☆^

**DOI:** 10.1016/j.psj.2025.105639

**Published:** 2025-08-06

**Authors:** Leonie Meier, Nadine Wallauch, Martina Feger, Michael Oster, Vera Sommerfeld, Sonja Schmucker, Klaus Wimmers, Korinna Huber, Volker Stefanski, Markus Rodehutscord, Michael Föller

**Affiliations:** a Department of Physiology, University of Hohenheim, 70599 Stuttgart, Germany; b Institute of Animal Science, University of Hohenheim, 70599 Stuttgart, Germany; c Research Institute for Farm Animal Biology (FBN), 18196 Dummerstorf, Germany

**Keywords:** Egg, Gene expression, Laying hen, Phosphorus, FGF23

## Abstract

Maintenance of phosphate homeostasis is particularly critical in laying hens for bone formation and calcium mobilization. The supplementation of their feed with mineral phosphate is common although recent research questions the usual levels of supplementation. Phosphate homeostasis is classically regulated by active vitamin D (calcitriol) and parathyroid hormone, whereas fibroblast growth factor 23 (FGF23) and its co-receptor αKlotho are novel factors. FGF23 has emerged as an important disease biomarker and αKlotho as an anti-aging factor in mammals, however, little is known about their role in poultry.

Here, we studied FGF23 and αKlotho expression in two commercial laying hen strains under conditions of dietary mineral phosphorus renunciation and sufficient phosphorus supply. Fifteen- and 20-week-old Lohmann Brown-Classic (LB) or LSL-Classic (LSL) hens were fed a standard maize-soybean-based diet containing 0 or 1 g/kg additional mineral phosphorus for 4 weeks. The animals were sacrificed, and gene expression studied in different organs by quantitative real-time PCR and protein expression by western blotting. Statistical correlation with further parameters of mineral metabolism was analyzed by Pearson’s correlation coefficient or Spearman’s Rho.

As a result, FGF23 bone expression was significantly lower and hepatic FGF23 expression higher in 24-week-old than in 19-week-old hens. Bone, hepatic, and renal αKlotho expression was significantly higher in older than younger animals. Compared to LB hens, LSL hens exhibited higher hepatic αKlotho irrespective of diet and age. Dietary phosphorus content did not significantly affect FGF23 and αKlotho expression. Bone FGF23 expression was positively and hepatic FGF23 negatively associated with plasma phosphate concentration whereas bone FGF23 expression was negatively and hepatic FGF23 positively associated with plasma calcitriol concentration.

To conclude, we uncovered a strong impact of age and strain on FGF23 and αKlotho expression in two high performance laying hen strains, effects possibly associated with initiation of the egg-laying phase. Moreover, the regulation of hepatic FGF23 expression differed from the regulation of bone FGF23 expression. Further studies are needed to elucidate the physiological relevance.

## Introduction

Laying hens are critically dependent on sufficient dietary phosphorus intake, particularly for bone formation (Rodehutscord et al., 2023; Whitehead, 2004). Classically, phosphate – and calcium – metabolism are subject to hormonal regulation by active vitamin D (calcitriol, 1,25(OH)_2_D_3_) and parathyroid hormone (**PTH**): Calcitriol enhances intestinal absorption of phosphate (Alexander, 2021) and calcium (Areco et al., 2020), thereby elevating serum phosphate and calcium levels (Bennin et al., 2024; Lang et al., 2018), whereas PTH has opposing effects on calcium and phosphate: In response to low serum calcium levels, i.e. hypocalcemia, PTH is released, increasing serum calcium concentration mainly by promoting bone degradation (Yu et al., 2020) and reducing urinary calcium excretion while lowering serum phosphate concentration (Alexander und Dimke, 2023; McKenna et al., 2021). The latter effect is mainly due to enhanced urinary phosphate excretion by inhibiting phosphate reabsorption in the kidney (McKenna et al., 2021).

First discovered in rodents 25 years ago, fibroblast growth factor 23 (**FGF23**) has emerged as an additional powerful endocrine regulator of phosphate homeostasis (Agoro und White, 2023). In mammals, FGF23 lowers renal phosphate reabsorption and calcitriol synthesis (Gattineni et al., 2009; Shimada et al., 2005), effects dependent on renal transmembrane αKlotho protein (Chen et al., 2018; Lindberg et al., 2014). Also in birds, including laying hens, FGF23 (Ren et al., 2017, 2020) and αKlotho (Ren et al., 2020; Wang et al., 2018) are regulators of mineral metabolism. However, whereas bone is the main source for FGF23 under physiological conditions in mammals (Mirams et al., 2004), hepatic FGF23 production is predominant in birds, with bone following as the second most important production site (Wang et al., 2018). Compared to mammals, considerably less is known about the regulation of FGF23 in laying hens. Similar to mammals, FGF23 is a major regulator of vitamin D metabolism (Gloux et al., 2020a) and phosphate excretion in laying hens (Ren et al., 2017). For regulation of serum phosphate concentration, FGF23 seems to be particularly relevant in broilers during dietary phosphorus excess (Magnuson et al., 2024). High phosphorus feeding enhances bone, but not hepatic FGF23 expression in laying hens (Wang et al., 2018). Vitamin D deficiency lowers FGF23 serum levels through reduced serum phosphate concentration in laying hens (Yan et al., 2022). Aging of laying hens is paralleled by enhanced FGF23 expression and lower serum calcitriol concentration (Gloux et al., 2020b). Also, the egg shell gland is a target of FGF23 as it expresses FGF23 receptors (Sinclair-Black et al., 2024).

Commonly, commercial laying hens are fed diets with additional mineral phosphorus to avoid any phosphate deficiency (Jing et al., 2018) whereas a recent summary of the literature indicated that the phosphorus requirement of laying hens is generally over-estimated (Rodehutscord et al., 2023). Plant-based laying hen feed is rich in phosphate in the form of phytate (Selle und Ravindran, 2007), the utilization of which may be low since laying hens have low endogenous activity of phytases, enzymes breaking down phytate, in the digestive tract (Dersjant-Li et al., 2015; Ma et al., 2019). However, recent research uncovered that laying hens (Sommerfeld et al., 2024, 2020) and, even more so, broiler chickens (Imari et al., 2020; Omotoso et al., 2023) may enhance intestinal utilization of non-mineral phosphorus when challenged with feed low in mineral phosphorus.

Recently, an extensive study was conducted comparing two commercial laying hen strains fed diets with or without mineral phosphorus supplementation before and after the onset of egg-laying activity (Sommerfeld et al., 2024). The objective of the present study was to explore the expression of FGF23 and αKlotho in view of their roles in phosphorus and calcium homeostasis. The study was complementary to measures of phosphate and calcium homeostasis parameters related to phosphorus supplementation, hen strain and age in the same animals (Qasir et al., 2025; Sommerfeld et al., 2024).

## Materials and methods

This study is part of the interdisciplinary Research Unit P-Fowl: Inositol phosphates and *myo*-inositol in the domestic fowl: Exploring the interface of genetics, physiology, microbiome, and nutrition (https://p-fowl.uni-hohenheim.de/). The experiment was conducted in accordance with German federal law and approved by the state of Baden-Württemberg (Project no. HOH67-21TE). Animals were kept at the Agricultural Experimental Station of the University of Hohenheim (Unterer Lindenhof, Eningen, Germany).

### Experimental setup and sampling

The experimental setup was described in detail by Sommerfeld et al., 2024. Briefly, the factors “hen strain”, “diet” and “period” made up a 2 × 2 × 2-factorial design with 10 hens in each group. Forty Lohmann Brown-Classic (**LB**) and 40 Lohmann LSL-Classic (**LSL**) hens provided by Lohmann Tierzucht GmbH (Cuxhaven, Germany) were investigated. The experiments consisted of two experimental periods: At 15 and 20 weeks of age, hens were placed in individual metabolic units (1 m x 1 m x 1 m) for 4 weeks. During this time, they were either fed a diet with additional 1 g mineral phosphorus/kg feed in the form of monocalcium phosphate (**P+**) or without it (**P-**). Diets contained all other nutrients as recommended by the Gesellschaft für Ernährungsphysiologie (Gesellschaft, 1999) and were without phytase supplements. Hen strain and diet allocation were randomized in a block design. Depending on the developmental stage, hens of the first period were fed different diets: a developer diet from weeks 15 to 16, a prelayer diet from weeks 16 to 17 and a layer diet from week 17 until sampling in week 19, either with or without supplemental phosphate (Sommerfeld et al., 2024). Hens of the second period were fed the layer feed from week 20 until sampling in week 24 either with or without supplemental inorganic phosphate.

Tissue sampling was conducted at 19 and 24 weeks of age. Hens were anesthetized with a gas mixture of 35 % CO_2_, 35 % N_2_ and 30 % O_2_ and decapitated. The right kidney was freed of urethra, chopped into small pieces, and immediately put on dry ice. Excess muscle and connective tissue was removed from the tibia, epiphyses were cut off, and tibiae were cut to generate a proximal and distal half. Bone marrow and medullary bone were removed by rinsing with 0.9 % ice-cold saline. After cleaning, parts of the tibia were immediately put on dry ice. A piece of the liver middle lobe was snap-frozen in liquid nitrogen, cut into pieces, and stored on dry ice. All samples were stored at −70°C until further use.

### RNA isolation and quantitative real-time PCR for FGF23 and αKlotho

For RNA isolation, kidney samples and the proximal part of the tibia were ground into powder with mortar and pestle in liquid nitrogen. Kidney and liver samples were homogenized in peqGOLD Trifast (Peqlab, VWR, Darmstadt, Germany) and bone samples in TRI reagent (Thermo Fisher Scientific, Waltham, MA, USA). RNA concentration and purity (260/280 and 260/230 ratios) were determined on a NanoDrop 2000 Spectrometer (Thermo Fisher Scientific).

DNase treatment was carried out by using the DNA-free Kit (Thermo Fisher Scientific) according to the product guide in a total volume of 30 µl. First-strand cDNA was made from 800 ng RNA of tibia and 1.2 µg of hepatic and renal tissue with the GoScript Reverse Transcription System using random primers (Promega, Mannheim, Germany) according to the product guide on a Biometra TAdvanced thermal cycler (Analytik Jena, Jena, Germany).

Quantitative real-time PCR (**qRT-PCR**) was used to determine gene expression levels of FGF23 and αKlotho, as well as the housekeeping gene TATA-box binding protein (**TBP**). The final volume of 20 µl consisted of 2 µl cDNA, primers at a concentration of 0.25 pmol/µl (*KL, FGF23*) or 0.5 pmol/µl (*TBP*), 10 µl GoTaq qPCR MasterMix (Promega) and 6 or 7 µl DNase- and RNase-free water. Gene expression was analyzed on a CFX Connect Real-Time System (Bio-Rad Laboratories, Feldkirchen, Germany). Temperature profile for the qRT-PCR was 95°C for 2 min, followed by 40 cycles of 95°C for 10 s, annealing at primer-specific temperatures (*KL*: 61°C, *FGF23*: 58°C, *TBP*: 59°C) for 30 s, and 72°C for 25 s. Samples without reverse transcriptase treatment and a no-template control were used as controls. The following primers were used (5‘ → 3‘):

*KL* (107 bp): GGTAGACACAACTCCTGCCC and TGCGTTGGGAGGTGAAAACT;
*FGF23* (189 bp): ATGCTGCTTGTGCTCTGTATC and ACTGTAAATGGTTTGGTGAGG;
*TBP* (182 bp): TGTGTCCACGGTGAATCTTG and GTTCCTCGCTTTTTGCTCCT.

Transcript levels of αKlotho (*KL*) and *FGF23* were normalized to transcript levels of *TBP* and the 2^-ΔCt^ method was used for evaluation. The housekeeping gene *TBP* exhibited consistent expression, with cycle threshold (**Ct**) values not significantly affected by the treatments (*p* > 0.05).

### Biomark HD based quantitative real-time PCR for IL-1ß

For gene expression analysis, RNA was extracted from 100 mg of liver tissue using Trizol Reagent (Thermo Fisher Scientific) according to the manufacturer’s protocol. Samples were homogenized using steel beads on a FastPrepTM FP120 (Thermo Electron Corporation, Karlsruhe, Germany). RNA was dissolved in nuclease-free water and RNA concentration and purity was measured using a NanoDrop2000 Spectrophotometer (Thermo Fisher Scientific). In addition, the integrity of extracted RNA of a random but representative subset (including all treatments) was checked via gel electrophoresis (Aranda et al., 2012) and on a Qubit 4 using the Qubit RNA IQ Assay Kit (#Q33221; both from Thermo Fisher Scientific).

Primers for analysis of gene expression of interleukin-1β (**IL-1β**) (Dalgaard et al., 2015) and glyceraldehyde-3-phosphate dehydrogenase (**GAPDH**) (Borowska et al., 2019) as reference gene were selected from previous studies. The following primers were used (5‘ → 3‘):

*GAPDH* (150 bp): GAAGGCTGGGGCTCATCTG and CAGTTGGTGGTGCACGATG;
*IL1B* (93 bp): TCCTGGAGGAGGTTTTTGAG and AGGACTGTGAGCGGGTGTAG.

Confirmation of primer specificity was performed in a standard PCR using DreamTaq Green (Thermo Fisher Scientific) with the same conditions as used for final analysis (see Table S1, supplementary file). Standard agarose gel electrophoresis was used to visualize resulting PCR products, which were further purified and sequenced bidirectionally by Sanger Sequencing technique (performed by Microsynth, Balgach, Switzerland).

Gene expression analysis was performed on a Biomark HD (Standard Bio Tools, South San Francisco, CA, USA) according to manufacturer’s protocols. To remove remaining DNA from RNA samples, 2 µg RNA extract was digested using DNase I (Thermo Fisher Scientific). Reverse Transcription Master Mix (Standard Bio Tools) was used for cDNA synthesis, with the input of 1 µl DNase-treated RNA. A 15-cycle pre-amplification step was performed using 1.25 µl cDNA, Preamp Master Mix (Standard Bio Tools) and a pool of all primers used for the final qPCR runs. Primers were removed by exonuclease I digestion (New England Biolabs, Frankfurt am Main, Germany) and samples were diluted fivefold.

The final qPCR runs were performed on a 96.96 Dynamic Array™ Integrated Fluidic Circuits (**IFC**) for Gene Expression (Standard Bio Tools) using Delta Gene Assays protocol with the manufacturer’s standard protocol for fast PCR and melting curve as described in Table S2 (supplementary file). IFC sample inlets were loaded with 5 µl of exo I-treated, pre-amplified and diluted sample mixed with SsoFast™ EvaGreen® Supermix with Low ROX (Bio-Rad Laboratories) and DNA Binding Dye Sample Loading Reagent (Standard Bio Tools). Primers were diluted with Assay Loading Reagent (Standard Bio Tools) and DNA Suspension Buffer (Thermo Fisher Scientific) to a final concentration of 5 μM and 5 µl of each primer dilution was loaded in the assay inlets in triplicates. To test contamination of the primers and reagents, a negative control was included throughout all preparation steps.

Standard Bio Tools Real-Time PCR analysis software (version 1.0.2) was used for data evaluation and quality control. The peak ratio threshold was set to 0.8 and quality threshold to 0.65. Means of triplicates were calculated for all samples. For samples that only had two or one successful triplicate, this respective run was used. The Ct values of IL-1β were normalized to the Ct values of *GAPDH* using the 2^-ΔCt^ method.

### Western blotting

Kidney and bone tissues were ground in the presence of liquid nitrogen to a fine powder, whereas liver samples were immediately subjected to homogenization by an tissue lyzer, and proteins were extracted using Tissue Protein Extraction Reagent (T-PER; Thermo Fisher Scientific) supplemented with a protease and phosphatase inhibitor cocktail and EDTA (Thermo Fisher Scientific). The reagent was applied at 20 µl per mg of tissue for liver and kidney, and 5 µl per mg for bone. Protein concentrations were determined using the Bradford assay (Thermo Fisher Scientific). For Western blot analysis, 30 µg of protein was loaded for liver and kidney samples, and 8 µg for bone samples. Proteins were separated using 10 % (for αKlotho) and 12 % (for FGF23) SDS-PAGE gels and transferred to membranes for detection of αKlotho and FGF23. Membranes were stained with Ponceau S solution (AppliChem, Darmstadt, Germany) and imaged with the ChemiDoc MP Imaging System (Bio-Rad Laboratories) for total protein normalization. The following primary antibodies were used: rat anti-FGF23 (1:1000, #MAB26291, R&D Systems, Minneapolis, MN, USA), rabbit anti-Klotho (1:1000, #LS-C107624, LS Bio, Vector Laboratories, Mowry Ave Newark, CA, USA) and mouse anti-ß-actin (1:10000, #A5441, Sigma-Aldrich, Schnelldorf, Germany). As secondary antibodies, anti-rabbit (#7074, Cell Signaling Technology, Danvers, MA, USA), anti-mouse (ab205719, abcam, Cambridge, UK) and anti-rat (#NBP1-75388, Novus Biologicals, Centennial, CO, USA) IgGs were used. Following detection of FGF23, membranes were stripped using ROTI®Free Stripping Buffer 2.2 plus (Carl Roth, Karlsruhe, Germany) for 30 minutes, according to the manufacturer's instructions, and subsequently re-probed with anti-β-actin. Protein bands were visualized using an ECL detection reagent and imaged with the ChemiDoc MP Imaging System (Bio-Rad Laboratories). Protein expression levels were quantified using Image Lab software (version 6.2; Bio-Rad Laboratories). Liver and tibia samples were normalized to loading control β-actin, while kidney samples were normalized to total protein staining.

### Analysis of plasma parameters

Methodology and data analysis of plasma parameters was already described in detail and published in Qasir et al., 2025.

### Statistical analysis

Kolmogorov-Smirnov test was used to test for normality. Data that were not normally distributed were transformed with Box-Cox transformation. Comparisons were performed with the MIXED procedure and pairwise *t*-tests on the statistical software SAS (version 9.3; SAS Institute, Cary, NC, USA). The following statistical model was used:

Y=strain+productionperiod+diet+strain*productionperiod+strain*diet+productionperiod*diet+strain*productionperiod*diet+father+block+ε

with Y as relative gene or protein expression, ε as residual error, strain, production period and diet as fixed effects, and father and block as random effects. An individual hen counted as an experimental unit. Results were presented as means ± standard error of mean of untransformed data. For correlation analysis, Pearson’s correlation for normally distributed data and Spearman’s rank correlation for not normally distributed data was used. Statistical significance was declared at *P* < 0.05.

## Results

Our study aimed to explore the impact of hen strain, age, and dietary phosphorus supply on FGF23 and αKlotho gene expression. Parameters of mineral metabolism (plasma inorganic phosphate, calcium, parathyroid hormone, calcidiol, and calcitriol) have already been published (Qasir et al., 2025) and are again illustrated in Supplementary Fig. S1. We determined FGF23 mRNA expression in the two tissues previously shown to exhibit the highest expression levels of FGF23 in birds (Wang et al., 2018), i.e. liver and bone (tibia) using qRT-PCR, followed by measurements of FGF23 protein expression in liver and tibia via western blotting.

**Fig. 1:**
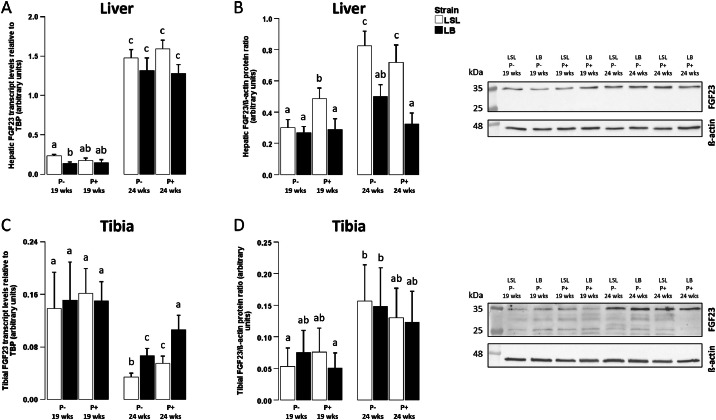
FGF23 mRNA and protein expression in liver and tibia in laying hens before and after onset of laying activity.

We found significantly higher hepatic FGF23 expression in LSL compared to LB hens at mRNA and protein level (mRNA: *P* = 0.001; protein: *P* < 0.001; Fig. 1A, 1B). Whereas dietary phosphorus content did not significantly affect hepatic FGF23 levels (mRNA: *P* = 0.479; protein: *P* = 0.658), the production period had a distinct effect (mRNA and protein: *P* < 0.001): Compared to week 19, hepatic FGF23 gene and protein expression was strongly upregulated at week 24 in either strain (Fig. 1A, 1B).

Hepatic (A,B) and bone (C,D) FGF23 gene expression (A,C) or protein abundance (B,D) in two different laying hen strains (Lohmann LSL (LSL; white) or Lohmann Brown (LB; black)) before (19 weeks) and after (24 weeks) onset of lay and fed with 1 g/kg (P+) or without (P-) supplemental mineral phosphorus. FGF23 gene expression (n = 10) is given as arbitrary units with TATA-box binding protein (TBP) as internal control. Values are presented as arithmetic means ± standard error of mean. For FGF23 protein, representative Western blots and arithmetic means ± standard error of mean of FGF23 protein expression (⁓ 35 kDa) in liver (B, n = 10) and tibia (D, n = 5) as arbitrary units with ß-actin (⁓ 42 kDa) as loading control are shown. Different superscripts indicate statistical differences between the experimental groups.

FGF23 expression in tibia was not significantly different between LSL hens and LB hens (mRNA: *P* = 0.068; protein: *P* = 0.924; Fig 1C, 1D). Similar to hepatic FGF23 expression, FGF23 in bone was not significantly affected by dietary phosphorus content (mRNA: *P* = 0.054; protein: *P* = 0.481; Fig. 1C, 1D). In contrast to hepatic FGF23 expression, however, FGF23 mRNA expression in bone was lower at week 24 compared to week 19 (*P* < 0.001; Fig. 1C). At protein level, however, tibial FGF23 was higher at week 24 than at week 19 (*P* = 0.001; Fig. 1D).

**Fig. 2:**
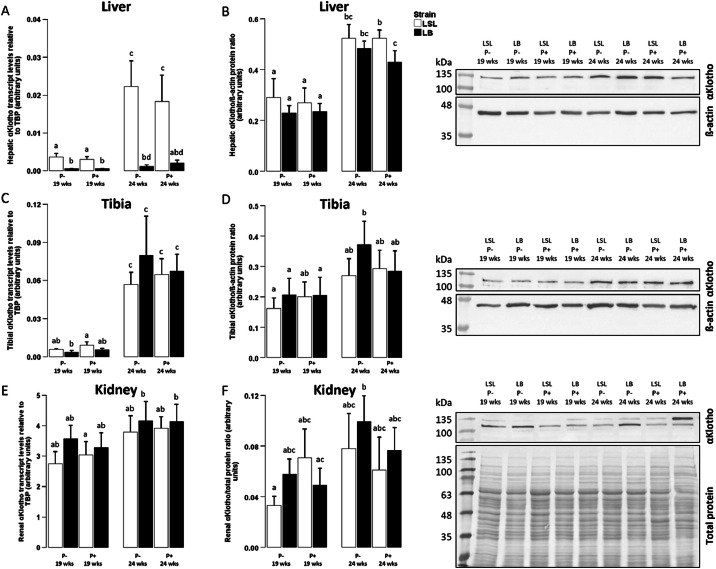
αKlotho mRNA and protein expression in liver, tibia and kidney in laying hens before and after onset of lay.

Hepatic αKlotho mRNA expression was markedly upregulated in LSL hens compared to LB hens (mRNA: *P* < 0.001) and at week 24 compared to week 19 (mRNA: *P* < 0.001) but was not dependent on dietary phosphorus content (mRNA: *P* = 0.958; Fig. 2A). Also, hepatic αKlotho protein abundance was higher at week 24 compared to week 19 (protein: *P* < 0.001; Fig. 2B). Dietary phosphorus content did not significantly affect αKlotho protein, either (protein: *P* = 0.428; Fig. 2B). Also, αKlotho protein was not significantly different between LSL and LB animals (protein: *P* = 0.981; Fig. 2B).

Hepatic (A,B), bone (C,D) and renal (E,F) αKlotho gene expression (A,C,E) or protein abundance (B,D,F) in two different laying hen strains (Lohmann LSL (LSL; white) or Lohmann Brown (LB; black)) before (19 weeks) and after (24 weeks) onset of lay and fed with 1 g/kg (P+) or without (P-) supplemental mineral phosphorus. For gene expression, results are presented as arbitrary units with TATA-box binding protein (TBP) as internal control. Values are presented as arithmetic means ± standard error of mean (n = 10). Representative Western blots and arithmetic means ± standard error of mean of αKlotho protein expression (⁓ 130-135 kDa) in liver (B, n = 10), tibia (D, n = 10) and kidney (F, n = 8) as arbitrary units with ß-actin (⁓ 42 kDa, for liver and tibia) and total protein (for kidney) as loading controls are shown. For renal αKlotho protein, analysis of bands at 130 or 135 kDa yielded no difference, hence, both bands were analyzed. Different superscripts indicate statistical differences between the experimental groups.

As illustrated in Fig. 2C, tibial αKlotho gene expression was slightly but significantly lower in LSL hens than LB hens at mRNA level (mRNA: *P* = 0.039). αKlotho protein was, however, not significantly different between the strains (protein: *P* = 0.311; Fig. 2D). Dietary phosphorus supply did not significantly affect tibia αKlotho expression (mRNA: *P* = 0.353; protein: *P* = 0.839), which was significantly higher at week 24 than 19 at both, mRNA and protein level (mRNA: *P* < 0.001; protein: *P* = 0.002; Fig. 2C, 2D).

Dietary phosphorus supply (mRNA: *P* = 0.606; protein: *P* = 0.865) and strain (mRNA: *P* = 0.282; protein: *P* = 0.468) did not significantly affect renal αKlotho mRNA or protein abundance (Fig. 2E, 2F). Compared to week 19, αKlotho gene expression was significantly higher at week 24 at mRNA and protein level (mRNA: *P* = 0.002; protein: *P* = 0.036; Fig. 2E, 2F).

**Fig. 3:**
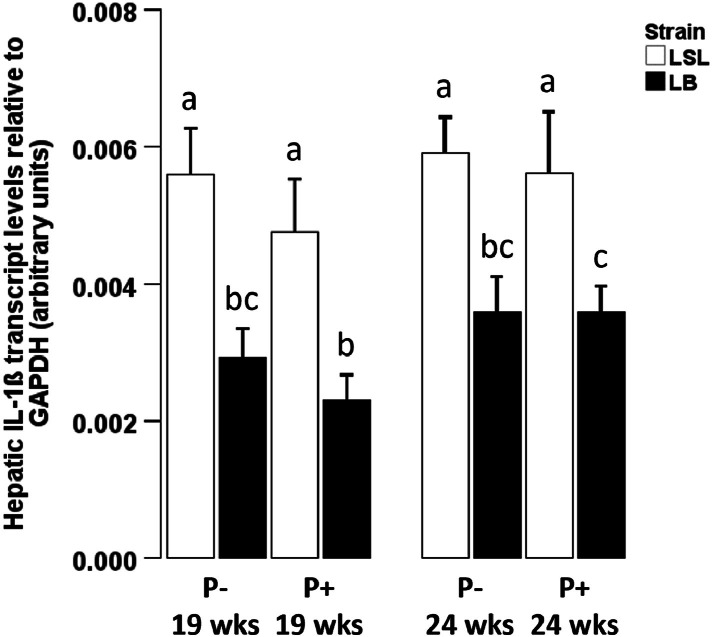
Hepatic mRNA IL-1ß expression before and after onset of laying activity.

To further estimate whether hepatic inflammation is also relevant for the regulation of FGF23 and αKlotho in laying hens as it is in mammals (Chi et al., 2023; Singh et al., 2016), hepatic expression of IL-1ß was measured. Dietary phosphorus did not influence hepatic IL-1ß expression (Fig. 3; *P* = 0.257). Compared to LB animals, IL-1ß expression was higher in LSL hens (*P* < 0.001) and higher in 24-week-old compared to 19-week-old hens (*P* = 0.042; Fig. 3). Additionally, we analyzed the association of hepatic IL-1ß expression with hepatic FGF23 and αKlotho expression. Taking both strains together, IL-1ß and FGF23 were positively correlated in animals fed P+ but not P- diet (P+: ρ = 0.338, *P* = 0.048; P-: ρ = 0.162, *P* = 0.355; Table 1). A positive correlation between IL-1ß and liver αKlotho was found in both diets (P+: ρ = 0.397, *P* = 0.032; P-: ρ = 0.575, *P* < 0.001; Table 1).

Hepatic interleukin-1β (IL-1ß) expression in two different laying hen strains (Lohmann LSL (LSL; white) or Lohmann Brown (LB; black)) before (19 weeks) and after (24 weeks) onset of lay and fed with 1 g/kg (P+) or without (P-) supplemental mineral phosphorus. Results are presented as arbitrary units with glyceraldehyde-3-phosphate dehydrogenase (GAPDH) as internal control and given as arithmetic means ± standard error of mean (*n* = 10). Different superscripts indicate statistical differences between the experimental groups.

**Table 1:** Correlation matrix of IL-1ß, FGF23 and αKlotho expression levels in liver, separated for dietary phosphorus levels.

	Liver IL-1ß expression [a.u.]
*P*+	
Liver FGF23 expression [a.u.]	0.338*
Liver αKlotho expression [a.u.]	0.397*
*P-*	
Liver FGF23 expression [a.u.]	*0.162*
Liver αKlotho expression [a.u.]	*0.575*⁎⁎⁎
	

Spearman’s rank correlation coefficient for not normally distributed data or Pearson’s correlation coefficient for normally distributed data.

⁎ *P* < 0.05,

⁎⁎⁎ *P* < 0.001 indicate significant correlation coefficients. Abbreviations: a.u. = arbitrary units, FGF23 = fibroblast growth factor 23, IL-1ß = interleukin-1ß, *P*+ = diet with 1g/kg supplemented mineral phosphorus, P- = diet without supplemented mineral phosphorus.

Lastly, the association of FGF23 and αKlotho expression with parameters of mineral metabolism obtained in a companion study (Qasir et al., 2025) was analyzed. Tibia FGF23 expression was positively correlated with plasma phosphate levels in both strains (LSL: ρ = 0.709, *P* < 0.001; LB: ρ = 0.707, *P* < 0.001; Table 2) and negatively associated with plasma calcitriol concentration only in LSL hens (LSL: ρ = −0.533, *P* = 0.004; LB: ρ = −0.244, *P* = 0.211; Table 2). Hepatic FGF23 expression was, however, inversely correlated with plasma phosphate concentration (LSL: ρ = −0.618, *P* < 0.001; LB: ρ = −0.693, *P* < 0.001) and positively associated with plasma calcitriol (LSL: ρ = 0.537, *P* < 0.001; LB: ρ = 0.602, *P* < 0.001) in both strains (Table 2). In a separate analysis of hens receiving feed with or without mineral phosphorus supplements, tibial FGF23 expression was again positively correlated (P+: ρ = 0.776, *P* < 0.001; P-: ρ = 0.657, *P* < 0.001), and hepatic FGF23 expression negatively correlated with plasma phosphate concentration (P+: ρ = −0.775, *P* < 0.001; P-: ρ = −0.724, *P* < 0.001; Table 3).

**Table 2:** Correlation matrix of plasma parameters and FGF23 expression levels in tibia and liver and αKlotho expression levels in tibia, liver, and kidney, separated for hen strain.

	Plasma inorganic P^1^ [mg/dl]	Plasma total calcium^1^ [mg/dl]	Plasma calcidiol^1^ [ng/ml]	Plasma calcitriol^1^ [pmol/l]	Plasma PTH^1^ [pg/ml]
*LSL hens*					
Tibia FGF23 expression [a.u.]	*0.709*⁎⁎⁎	*−0.516*⁎⁎	*0.539*⁎⁎	*−0.533*⁎⁎	0.438*
Liver FGF23 expression [a.u.]	*−0.618*⁎⁎⁎	*0.816*⁎⁎⁎	−0.362*	*0.537*⁎⁎⁎	*−0.574*⁎⁎⁎
Tibia αKlotho expression [a.u.]	−0.442*	*0.683*⁎⁎⁎	*−0.130*	0.473*	*−0.634*⁎⁎⁎
Liver αKlotho expression [a.u.]	*−0.536*⁎⁎⁎	*0.734*⁎⁎⁎	*−0.114*	0.330*	*−0.520*⁎⁎⁎
Kidney αKlotho expression [a.u.]	*−0.287*	*0.207*	*−0.029*	*0.485*⁎⁎	−0.342*
*LB hens*					
Tibia FGF23 expression [a.u.]	*0.707*⁎⁎⁎	*−0.242*	*0.080*	*−0.244*	*0.122*
Liver FGF23 expression [a.u.]	*−0.693*⁎⁎⁎	*0.764*⁎⁎⁎	*−0.151*	*0.602*⁎⁎⁎	*−0.164*
Tibia αKlotho expression [a.u.]	*−0.572*⁎⁎	*0.665*⁎⁎⁎	*−0.305*	0.426*	*−0.215*
Liver αKlotho expression [a.u.]	*−0.217*	0.375*	*−0.234*	*0.209*	*−0.142*
Kidney αKlotho expression [a.u.]	*−0.310*	*0.062*	*0.262*	*0.084*	*0.297*

Spearman’s rank correlation coefficient for not normally distributed data or Pearson’s correlation coefficient for normally distributed data.

⁎ *P* < 0.05,

⁎⁎ *P* < 0.01,

⁎⁎⁎ *P* < 0.001 indicate significant correlation coefficients. Abbreviations: a.u. = arbitrary units, FGF23 = fibroblast growth factor 23, LB = Lohmann Brown-Classic, LSL = Lohmann LSL-Classic, P = phosphate, PTH = parathyroid hormone.

1 Data from Qasir et al., 2025.

**Table 3:** Correlation matrix of plasma parameters and FGF23 expression levels in tibia and liver and αKlotho expression levels in tibia, liver and kidney, separated for dietary phosphorus level.

	Plasma inorganic P^1^ [mg/dl]	Plasma total calcium^1^ [mg/dl]	Plasma calcidiol^1^ [ng/ml]	Plasma calcitriol^1^ [pmol/l]	Plasma PTH^1^ [pg/ml]
*P+*					
Tibia FGF23 expression [a.u.]	*0.776*⁎⁎⁎	*−0.269*	*0.309*	−0.401*	*0.348*
Liver FGF23 expression [a.u.]	*−0.775*⁎⁎⁎	*−0.774*⁎⁎⁎	*−0.280*	*0.559*⁎⁎⁎	−0.320*
Tibia αKlotho expression [a.u.]	*−0.718*⁎⁎⁎	*0.623*⁎⁎⁎	*−0.182*	*0.489*⁎⁎	*−0.373*
Liver αKlotho expression [a.u.]	*−0.566*⁎⁎⁎	0.342*	*−0.689*⁎⁎⁎	*0.428*⁎⁎	*−0.302*
Kidney αKlotho expression [a.u.]	*−0.288*	*0.258*	*0.287*	*0.240*	*0.071*
*P-*					
Tibia FGF23 expression [a.u.]	*0.657*⁎⁎⁎	*−0.332*	*0.479*⁎⁎	−0.476*	*0.358*
Liver FGF23 expression [a.u.]	*−0.724*⁎⁎⁎	*0.745*⁎⁎⁎	−0.324*	*0.624*⁎⁎⁎	*−0.487*⁎⁎
Tibia αKlotho expression [a.u.]	*−0.551*⁎⁎	*0.706*⁎⁎⁎	*−0.188*	*0.484*⁎⁎	−0.455*
Liver αKlotho expression [a.u.]	−0.369*	*0.259*	*−0.635*⁎⁎⁎	*0.195*	*−0.153*
Kidney αKlotho expression [a.u.]	−0.377*	*0.087*	*0.164*	0.424*	*−0.319*

Spearman’s rank correlation coefficient for not normally distributed data or Pearson’s correlation coefficient for normally distributed data.

⁎ *P* < 0.05,

⁎⁎ *P* < 0.01,

⁎⁎⁎ *P* < 0.001 indicate significant correlation coefficients. Abbreviations: a.u. = arbitrary units, FGF23 = fibroblast growth factor 23, P = phosphate, P+ = diet with 1g/kg supplemented mineral phosphorus, P- = diet without supplemented mineral phosphorus, PTH = parathyroid hormone.

1 Data from Qasir et al., 2025.

## Discussion

This study sought to explore whether dietary phosphorus content, laying phase or strain had significant effects on FGF23 and αKlotho expression in relevant organs of laying hens. First, it was found that a lack of supplemental phosphorus in laying hens did not significantly affect FGF23 or αKlotho expression in all tissues studied. Secondly, hepatic FGF23 gene expression was higher and tibia FGF23 gene expression lower in older than younger laying hens. Additionally, hepatic, renal, and tibial αKlotho expression was strongly upregulated in 24-week-old compared to 19-week-old hens. LSL hens exhibited higher hepatic FGF23 and αKlotho expression than LB hens, while LB hens showed higher renal αKlotho expression. We did not determine renal FGF23 expression since it is low compared to hepatic and tibial expression (Wang et al., 2018). Qasir et al., 2025 did not detect FGF23 expression through RNA sequencing in renal tissue, either.

Our study is complementary to studies by Sommerfeld et al., 2024 and Qasir et al., 2025. Sommerfeld et al., 2024 found out that a renunciation of phosphorus leads to increased endogenous phytate degradation while hens fed a diet supplemented with 1 g mineral phosphorus/kg feed retain more phosphorus. Differences in phosphorus metabolism before and after onset of lay may be attributed to different calcium needs in these periods. Qasir et al., 2025 focused on the effect of different dietary mineral phosphorus levels, different strain and sexual maturation on plasma parameters of phosphorus metabolism and kidney expression of genes related to phosphorus utilization. According to this study, plasma phosphate decreased and plasma calcium increased after the onset of lay. Both strains showed distinct compensatory mechanisms to deal with phosphorus renunciation, which were more pronounced in week 19, compared to week 24.

The analysis of hepatic FGF23 protein abundance confirmed the qRT-PCR results (age and strain effect). In contrast, FGF23 mRNA expression declined with the onset of lay, whereas FGF23 protein levels increased in bone. Further studies in laying hens are required to substantiate this hypothesis.

In some cases, significant differences in αKlotho gene expression could not be confirmed by significant differences in αKlotho protein abundance as determined by western blotting. It has to be kept in mind that subtle differences in gene expression may not affect protein expression. Moreover, it is possible that western blotting cannot resolve the discrete differences in αKlotho protein abundance in some cases.

A different FGF23 gene expression in bone and liver of hens has previously been observed by Wang et al., 2018, who found that bone FGF23, but not hepatic FGF23 expression is influenced by different levels of dietary phosphorus. In their study, 25-week-old laying hens were either fed 0.15 %, 0.40 % or 0.80 % available phosphorus for 11 days. Accordingly, tibia FGF23 expression may be more relevant for phosphate homeostasis than hepatic FGF23 expression (Wang et al., 2018). The role of hepatic FGF23 expression in laying hens has not been established yet. In mammals, hepatic FGF23 expression is enhanced only under pathophysiological conditions, such as acute or chronic liver injury (Jung et al., 2022; Kumar et al., 2022). In laying hens, the egg-laying period is associated with hepatic lipid accumulation, presumably inducing low-grade inflammation (Du et al., 2024; Miao et al., 2023). Particularly, high-yielding laying hens are at risk of developing fatty liver hemorrhagic syndrome (**FLHS**), characterized by excessive hepatic lipid accumulation, hepatic damage and inflammation (Shini et al., 2020). FLHS (Wang et al., 2025) and hepatic inflammation (Nii et al., 2020) are associated with higher expression of IL-1ß . In mammals, IL-1ß stimulates FGF23 production (McKnight et al., 2020). Conversely, in mammals FGF23 may also stimulate IL-1ß (Hu et al., 2024). We found that both, hepatic FGF23 and IL-1ß expression, were significantly higher in 24-week-old hens compared to 19-week-old hens. Importantly, FGF23 expression was positively associated with IL-1ß expression in hens fed the P+ diet. It is therefore intriguing to speculate that the increase in hepatic FGF23 expression was, at least in part, due to hepatic inflammation and increased IL-1ß expression.

The relationship between dietary phosphate levels and FGF23 has been controversial across studies in mammals (Antoniucci et al., 2006; Gutiérrez et al., 2011; Ito et al., 2007; Nishida et al., 2006) and poultry (Poorhemati et al., 2023). In our study, absence of mineral phosphorus supplementation had no significant effect on tibial or hepatic FGF23 expression compared to a diet with 1 g/kg supplemental mineral phosphorus. It is possible that the difference in available phosphorus between our diets was not large enough to induce an effect on FGF23 expression. Companion studies also found no disturbance of phosphorus homeostasis in the P- group (Qasir et al., 2025; Sommerfeld et al., 2024). Wang et al., 2018 showed that tibia FGF23 expression in 25-week-old hens was higher on a high phosphorus diet (0.80 % available phosphorus) compared to a low phosphorus diet (0.15 % available phosphorus), while there was no significant difference compared to a medium phosphorus diet (0.40 % available phosphorus). Ren et al., 2020 observed an increase in calvaria FGF23 expression on a higher phosphate diet (0.14 % of non-phytate phosphorus vs. 0.32 % non-phytate phosphorus) in 40-week-old hens. Our study addressed phosphorus renunciation in hens before and after the onset of lay and found that age did not change the effect of the diet. The excess of dietary phosphorus in the high phosphorus groups in Wang et al., 2018 and Ren et al., 2020 is higher than in our study. Possibly, an excess of dietary phosphorus ramps up bone FGF23 expression while a reduction of dietary phosphorus does not alter it. Another possible explanation is that dietary phosphorus levels were still not low enough to decrease FGF23 expression since mineral homeostasis was successfully maintained (Qasir et al., 2025; Sommerfeld et al., 2024). Indeed, recent studies indicate that the recommended dietary phosphorus content may actually be too high (Rodehutscord et al., 2023) and higher phytate usage by endogenous phosphatases, can be achieved if the diet is supplemented with neither dietary phosphorus nor exogenous phytase (Sommerfeld et al., 2024). Our finding that a phosphorus-reduced diet did not significantly affect FGF23 expression further supports the idea that laying hens require less mineral phosphorus than commonly believed. Also, the difference in dietary phosphorus supply between the groups may be too small to alter FGF23 expression levels.

The production period had the most pronounced effect on both, FGF23 and αKlotho expression, most likely reflecting physiological and metabolic changes in the process of sexual maturation. It is important to note that after week 17, laying hens received a layer diet consisting of more calcium (developer: 9.0 g/kg, prelayer: 22.5 g/kg, layer: 35.0 g/kg) with slight changes in dietary phosphorus (developer: 3.0/4.0 g/kg, prelayer: 3.3/4.3 g/kg, layer: 3.1/4.1 g/kg; Sommerfeld et al., 2024). This dietary difference is paralleled by a decrease in plasma phosphate levels in week 24 and an increase in plasma calcium levels (Omotoso et al., 2021; Qasir et al., 2025; Sommerfeld et al., 2024) which might be a driver for some age-related effects. Fast calcium mobilization for eggshell formation is accomplished by medullary bone that contains easily-accessible calcium and phosphate in the form of hydroxyapatite (Sinclair-Black et al., 2023; Whitehead, 2004). It undergoes daily alterations in calcium and phosphate concentrations (Kerschnitzki et al., 2014). Recent studies show that FGF23 expression in medullary bone (Gloux et al., 2020a) and the ratio of FGF23 expression of medullary bone relative to cortical bone (Hadley et al., 2016) are regulated by the time of oviposition. This may indicate that FGF23’s function in mineralization of medullary bone, but not cortical bone, is influenced by the daily egg-laying cycle. The observed decrease in cortical tibia FGF23 expression following egg laying may, therefore, reflect a functional shift in FGF23’s role in bone mineral metabolism. The simultaneous increase in hepatic FGF23 expression may also show that sites of FGF23 expression change with sexual maturation. Studies on intestinal gene expression have shown a focus shift between 19- and 24-week-old laying hens from immune function to energy metabolism: According to Shi et al., 2024, gene expression in the intestinal epithelium at 19 weeks favors immune function while at 24 weeks, egg production and energy metabolism are of higher priority. Similar observations were made by Abitew et al., 2024 demonstrating that immune cells and genes involved in the immune signaling pathways are more active and expressed at 19 weeks of age, while at 24 weeks, genes related to steroid biosynthesis and energy metabolism exhibited higher expression. The elevation of hepatic FGF23 expression upon the start of egg laying may, therefore, be associated with a higher inflammatory state in the chicken due to a shift away from immune signaling pathways, a hypothesis in line with the aforementioned possibility that hepatic FGF23 expression may particularly be influenced by liver inflammation. In line with this, we did observe an association of hepatic IL-1β expression with hepatic FGF23.

The positive correlation of tibia FGF23 expression with plasma phosphate is similar to the situation in mammals (Kobayashi et al., 2006; Ubaidus et al., 2009), suggesting that at least the role of bone FGF23 in phosphorus regulation in hens may be similar to that of mammals. We observed, however, differences between LSL and LB hens. In a companion study, LSL hens exhibited higher phosphate retention on a P- diet than LB hens or LSL hens with P+ diet (Sommerfeld et al., 2024). We observed that tibia FGF23 expression was positively correlated with plasma PTH and negatively with plasma calcitriol – irrespective of diet and age – in LSL hens only. Indeed, the interplay of PTH, calcitriol, and FGF23 in poultry has remained controversial: While one study in Hy-Line Brown laying hens reported no effect of FGF23 on plasma PTH and calcitriol (Ren et al., 2020), another study on Single Comb White Leghorns found that FGF23 increased plasma PTH and decreased plasma calcitriol (Ren et al., 2017). These contradicting results suggest that this hormonal crosstalk may be strain-dependent. Importantly, after the onset of egg laying, PTH appears crucial for intestinal calcium and phosphorus uptake, as well as eggshell formation (Garcia-Mejia et al., 2024). In view of the prioritization of calcium mobilization for eggshell calcification, FGF23’s suppressing effect on PTH, observed in mammals (Bergwitz und Jüppner, 2010; Komaba und Fukagawa, 2010), may be diminished in laying hens.

Irrespective of the diet, we observed a positive correlation between tibia FGF23 expression and plasma phosphate levels, supporting the view that the P- diet was sufficient to sustain phosphate homeostasis. In addition, it is possible that FGF23 is secreted to avoid an increase in plasma phosphate. Differences in the associations of tibia FGF23 expression and other plasma metabolites between the diet groups may be due to a higher need for phosphate uptake and reabsorption in the P- group.

### Conclusion

Renunciation of dietary mineral phosphorus did not significantly affect FGF23 or αKlotho expression levels in laying hens. The production period had a strong effect on FGF23 and αKlotho expression with tibial FGF23 levels decreasing and hepatic, renal, and tibial αKlotho levels and hepatic FGF23 levels increasing upon the onset of laying activity. Further studies are needed to clarify the underlying mechanisms, particularly the regulation of FGF23 and αKlotho and their potential role in inflammatory conditions such as fatty liver hemorrhagic syndrome.

## CRediT authorship contribution statement

**Leonie Meier:** Data curation, Formal analysis, Methodology, Visualization, Writing – original draft, Writing – review & editing. **Nadine Wallauch:** Data curation, Formal analysis, Methodology, Writing – review & editing. **Martina Feger:** Data curation, Formal analysis, Methodology, Supervision, Writing – review & editing. **Michael Oster:** Data curation, Funding acquisition, Methodology, Supervision, Writing – review & editing. **Vera Sommerfeld:** Conceptualization, Funding acquisition, Project administration, Supervision, Writing – review & editing. **Sonja Schmucker:** Conceptualization, Data curation, Funding acquisition, Methodology, Supervision, Writing – review & editing. **Klaus Wimmers:** Conceptualization, Funding acquisition, Methodology, Supervision, Writing – review & editing. **Korinna Huber:** Conceptualization, Funding acquisition, Supervision, Writing – review & editing. **Volker Stefanski:** Conceptualization, Data curation, Funding acquisition, Supervision, Writing – review & editing. **Markus Rodehutscord:** Conceptualization, Funding acquisition, Project administration, Supervision, Writing – review & editing. **Michael Föller:** Conceptualization, Funding acquisition, Investigation, Resources, Supervision, Writing – original draft, Writing – review & editing.

## Disclosures

The authors declare the following financial interests/personal relationships which may be considered as potential competing interests: Leonie Meier reports financial support was provided by 10.13039/501100001736German Research Foundation. Martina Feger reports financial support was provided by 10.13039/501100001736German Research Foundation. Michael Oster reports financial support was provided by 10.13039/501100001736German Research Foundation. Vera Sommerfeld reports financial support was provided by 10.13039/501100001736German Research Foundation. Klaus Wimmers reports financial support was provided by 10.13039/501100001736German Research Foundation. Korinna Huber reports financial support was provided by 10.13039/501100001736German Research Foundation. Markus Rodehutscord reports financial support was provided by 10.13039/501100001736German Research Foundation. Michael Foeller reports financial support was provided by 10.13039/501100001736German Research Foundation. Michael Foeller reports a relationship with Kyowa Kirin that includes: speaking and lecture fees. If there are other authors, they declare that they have no known competing financial interests or personal relationships that could have appeared to influence the work reported in this paper.

## Appendix Supplementary materials

Image, application 1
