# Supplementary material for: Expression of fibroblast growth factor 23 (FGF23) and αKlotho in two commercial laying hen strains fed with and without dietary mineral P supplements before and after the onset of the laying phase
Source: Poult Sci. 2025 Aug 6;104(11):105639. doi: 10.1016/j.psj.2025.105639 (PMC12395529; doi:10.1016/j.psj.2025.105639)
Supplement: Supplementary file 1 [file mmc1.docx]

***Supplementary data***

**Table S1:** PCR conditions used for all PCR reactions during evaluation of specificity, using Dream Taq (Thermo Fisher Scientific).

| **Temperature [°C]** | **Time** |  | |
| --- | --- | --- | --- |
| 95 | 2 min | |  |
| 95 | 30 sec | |  |
| 60 | 30 sec | | x 35 |
| 72 | 1 min | |  |
| 72 | 5 min | |  |
| 4 | ∞ | |  |

**Table S2:** Thermal cycling parameters used on the final qPCR runs according to the manufacturer’s protocol (Standard Bio Tools).

| Step | Flex Six | | | 96.96 IFC | | |
| --- | --- | --- | --- | --- | --- | --- |
|  | **Temperature [°C]** | **Time** | **Temperature [°C]** | | | **Time** |
| Thermal mixing | 25 | 30 min | 70 | | 40 min | |
|  | 70 | 60 min | 60 | | 30 sec | |
| Hot start | 95 | 1 min | 95 | | 1 min | |
| PCR, 30 cycles | 96 | 5 sec | 96 | | 5 sec | |
|  | 60 | 20 sec | 60 | | 20 sec | |
| Melting curve | 60 | 3 sec | 60 | | 3 sec | |
|  | 60-95 | 1°C/3 sec | 60-95 | | | 1°C/3 sec |

**Supplementary figure**

**
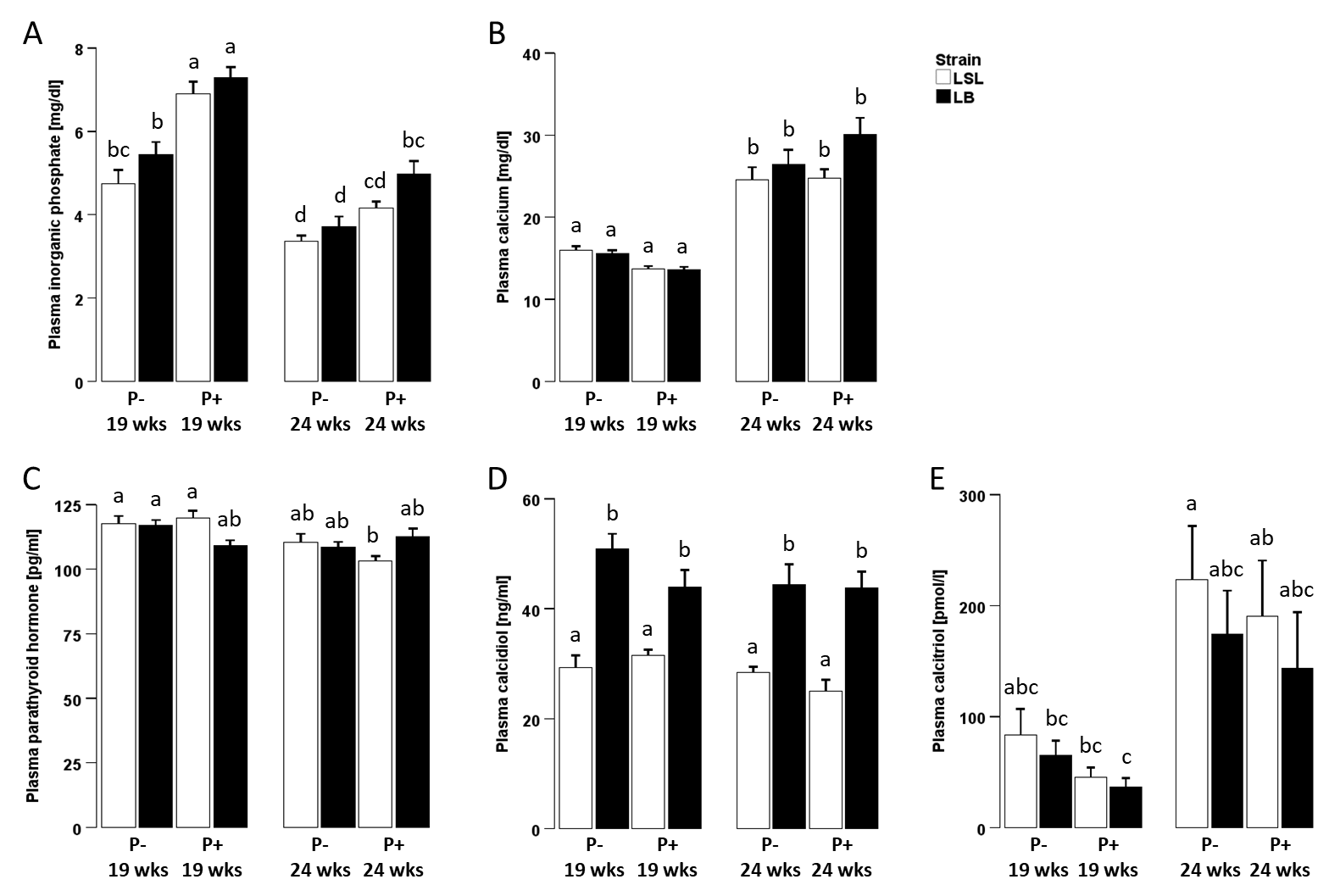
**

**Figure S1:** Plasma parameters for mineral homeostasis before and after onset of laying activity
Plasma levels of inorganic phosphate (A), total calcium (B), parathyroid hormone (C), calcidiol (D) and calcitriol (E) in laying hens before (19 weeks) and after (24 weeks) onset of lay fed with 1 g/kg (P+) or without (P-) supplemental alimentary phosphorus in two different laying hen strains (Lohmann LSL (LSL; white) or Lohmann Brown (LB; black)). Values are presented as arithmetic means ± standard error of mean. Data are already published in Qasir et al. 2025. Different superscripts indicate statistical differences between the experimental groups.

***Supplementary reference***

Qasir, Hiba; Reyer, Henry; Oster, Michael; Ponsuksili, Siriluck; Trakooljul, Nares; Sommerfeld, Vera et al. (2025): Effects of a transient lack of dietary mineral phosphorus on renal gene expression and plasma metabolites in two high-yielding laying hen strains. In: *BMC genomics* 26 (1), S. 129. DOI: 10.1186/s12864-025-11294-6.
